# Involvement of GTPases and vesicle adapter proteins in Heparan sulfate biosynthesis: role of Rab1A, Rab2A and GOLPH3

**DOI:** 10.1111/febs.17398

**Published:** 2025-01-13

**Authors:** Maria C. Z. Meneghetti, Renan P. Cavalheiro, Edwin A. Yates, Helena B. Nader, Marcelo A. Lima

**Affiliations:** ^1^ Departamento de Bioquímica, Instituto de Farmacologia e Biologia Molecular, Escola Paulista de Medicina Universidade Federal de São Paulo Brazil; ^2^ Department of Biochemistry, Cell and Systems Biology, Institute of Systems, Molecular and Integrative Biology University of Liverpool UK; ^3^ Centre for Glycoscience Keele University UK

**Keywords:** biosynthesis, GOLPH3, heparan sulfate, Rab1A, Rab2A

## Abstract

Vesicle trafficking is pivotal in heparan sulfate (HS) biosynthesis, influencing its spatial and temporal regulation within distinct Golgi compartments. This regulation modulates the sulfation pattern of HS, which is crucial for governing various biological processes. Here, we investigate the effects of silencing Rab1A and Rab2A expression on the localisation of 3‐O‐sulfotransferase‐5 (3OST5) within Golgi compartments and subsequent alterations in HS structure and levels. Interestingly, silencing Rab1A led to a shift in 3OST5 localization towards the trans‐Golgi, resulting in increased HS levels within 24 and 48 h, while silencing Rab2A caused 3OST5 accumulation in the cis‐Golgi, with a delayed rise in HS content observed after 48 h. Furthermore, a compensatory mechanism was evident in Rab2A‐silenced cells, where increased Rab1A protein expression was detected. This suggests a dynamic interplay between Rab1A and Rab2A in maintaining the fine balance of vesicle trafficking processes involved in HS biosynthesis. Additionally, we demonstrate that the trafficking of 3OST5 in COPI vesicles is facilitated by GOLPH3 protein. These findings identify novel vesicular transport mechanisms regulating HS biosynthesis and reveal a compensatory relationship between Rab1A and Rab2A in maintaining baseline HS production.

## Graphical abstract

Vesicle trafficking governs the biosynthesis of heparan sulfate (HS) by controlling the positioning of HS‐modifying enzymes within Golgi compartments. The proteins Rab1A and Rab2A play dynamic, compensatory roles that affect HS levels by influencing the transport of these enzymes. GOLPH3 facilitates the COPI vesicle‐mediated transport of HS‐modifying enzymes, revealing new mechanisms and interactions vital for maintaining balanced HS production, which is crucial for cellular functions.

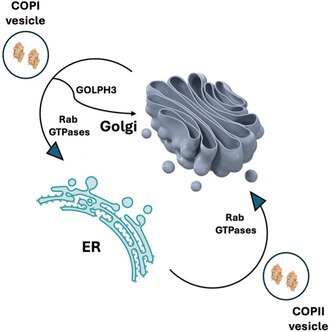

**Abbreviations**
2OST: 2‐O‐sulfotransferase
3OST5: 3‐O‐sulfotransferase‐5
6OST1: 6‐O‐sulfotransferase‐1
BSA: bovine serum albumin
DAPI: 4′,6‐diamidino‐2‐phenylindole
dp: degree of polymerization
EC: endothelial cell
EDTA: ethylenediaminetetraacetic acid
ER: endoplasmic reticulum
GFP: green fluorescent protein from *Aequorea coerulescens*
GlcA: D‐glucuronic acid
GlcNAc: N‐acetyl D‐glucosamine
GM130: Golgi matrix protein 130
GOLPH3: Golgi phosphoprotein 3
HS: heparan sulfate
IP: immunoprecipitation
NDST1: N‐deacetylase/N‐sulfotransferase‐1
NP‐40: nonidet P‐40
PBS: phosphate‐buffered saline
PDA: 1,3‐diaminepropane acetate
RIPA buffer: radioimmunoprecipitation assay buffer
Tris: tris(hydroxymethyl)aminomethane

## Introduction

Heparan sulfate (HS) is a complex polysaccharide found on the surface of many cells and in the extracellular matrix. It is involved in various biological processes, including cell signalling, development and disease. Its biosynthesis requires a series of enzymatic reactions within the endoplasmic reticulum (ER) and Golgi apparatus. The initial steps of HS biosynthesis as proteoglycans involve assembling an *O*‐linked tetrasaccharide linker region (xylose–galactose–galactose–glucuronic acid) utilizing a series of glycosyltransferases followed by the polymerization of a repeating disaccharide unit composed of 1,4‐linked β‐D‐glucuronic acid (GlcA) and N‐acetyl α‐D‐glucosamine (GlcNAc) residues by EXT1/EXT2 glycosyltransferases. This polymer backbone can then be further modified by specific HS‐modifying enzymes that do not result in complete HS substitution, resulting in complex substitution patterns [1, 2].

Biosynthetic models propose that HS‐modifying enzymes form complexes and act collectively [3, 4, 5], with reactions occurring in a hierarchical order [6, 7]. Other biosynthetic models also account for the relative abundance of both common and uncommon structures [8, 9, 10]. However, the final structure of HS is determined by the combined actions of both biosynthetic HS‐modifying enzymes and postsynthesis HS‐modifying enzymes, such as extracellular sulfatases. Therefore, understanding the trafficking and compartmentalization of these enzymes is crucial to elucidating the mechanisms of HS biosynthesis.

Vesicle trafficking plays a critical role in transporting HS biosynthetic enzymes between different compartments of the Golgi apparatus. Such a process requires spatial and temporal regulation, which has been shown to be critical for regulating HS biosynthesis [11, 12]. Specifically, enzymes involved in HS biosynthesis may be localized to different subcompartments within the Golgi depending on cellular status, where changes in the distribution of HS‐modifying enzymes correlate with the cell‐produced HS structure [12]. Notwithstanding its ubiquitous distribution and biological importance, the fundamental pathways underpinning the sorting and trafficking of its biosynthetic enzymes within the Golgi remain poorly defined.

The Rab protein family comprises a group of small GTPases that regulate vesicular transport and membrane trafficking in eukaryotic cells. Their primary function is to regulate intracellular vesicle targeting, fusion and recycling. Each Rab protein is localized to a specific membrane compartment and interacts with a unique set of effector proteins to coordinate the transport of cargo vesicles between organelles [13]. While there may be some overlap in the functions of different Rab proteins, there is not generally significant redundancy between them. Rather, the specificity of Rab protein function is thought to be crucial for ensuring proper targeting and fusion of vesicles to the correct subcellular compartment. For instance, Rab1A and Rab2A regulate intracellular vesicle trafficking and are localized to the ER–Golgi interface. Rab1A regulates the trafficking of proteins from the ER to the Golgi, while Rab2A regulates the trafficking of vesicles back to the ER [14, 15], all of which are known transport routes for HS biosynthetic enzymes [12].

The coat protein complexes I and II (COPI and COPII) facilitate the vesicle‐mediated protein transport from the endoplasmic reticulum to the Golgi apparatus or *vice versa* and within the different Golgi cisternae. The COPI adaptor GOLPH3 is essential for retaining a subset of glycosyltransferases within the Golgi apparatus by directly binding to the cytoplasmic tails of these enzymes [16]. At the Golgi membrane, GOLPH3 engages with both the COPI coat and the enzyme cargo, facilitating their packaging into vesicles destined for recycling from the trans‐Golgi network to the medial Golgi [17, 18].

Building upon our original research on how vesicle trafficking impacts HS biosynthesis, we anticipated that additional factors in the Golgi apparatus may regulate such a process. Consequently, we evaluated how Rab proteins, which mediate vesicular trafficking, influence HS‐modifying enzymes distribution. Furthermore, the first direct evidence that vesicle adaptor proteins, GOLPH3, bind to HS‐modifying enzymes is presented. Together, our approach brings new insight into how accessory proteins of coated vesicles regulate HS biosynthesis and how cells use intra‐Golgi complementary transport pathways to maintain baseline HS production.

## Results

### siRNA knockdown of Rab1A and Rab2A reveals a compensatory mechanism able to maintain Golgi structure

**Fig. 1:**
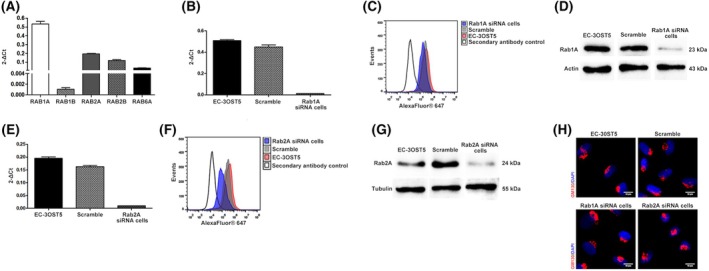
Knockdown of Rab1A and Rab2A in EC‐3OST5 cells. (A) Rab mRNA level in EC‐3OST5. siRNA‐silencing profile of Rab1A (B–D) and Rab2A (E–G). EC‐3OST5 cells were transfected with a mixture of three siRNA (10 nm) targeting each Rab, using lipofectamine 3000 (0.75 μL per well). Gene and protein expression analyses were conducted 48 h later. Rab gene expression level was analysed using real‐time PCR (B and E) and protein expression level was evaluated using flow cytometry (C and F) and western blotting (D and G). The results represent the mean and standard deviation (SD) from three independent experiments. (H) The structure of Golgi was assessed in siRNA transfected cells by confocal microscopy to GM130, the matrix protein of the cis‐Golgi, in two separate experiments. GM130 is shown in red and nuclei in blue. Scale bars: 10 μm.

We have previously shown that the trafficking of HS‐modifying enzymes mediated by COPI and COPII regulates HS biosynthesis [12]. Yet, the role of other vesicle accessory proteins in this process is unclear. Here, Rab1A and Rab2A proteins, essential accessory proteins in anterograde and retrograde transport, were transiently silenced with siRNA (Fig. S1 and Fig. 1B–G). Besides their fundamental role in vesicular transport between the ER–Golgi pathway, Rab1A and Rab2A also showed the highest mRNA expression levels amongst the Rab proteins evaluated in EC‐3OST5 cells (Fig. 1A).

**Fig. 2:**
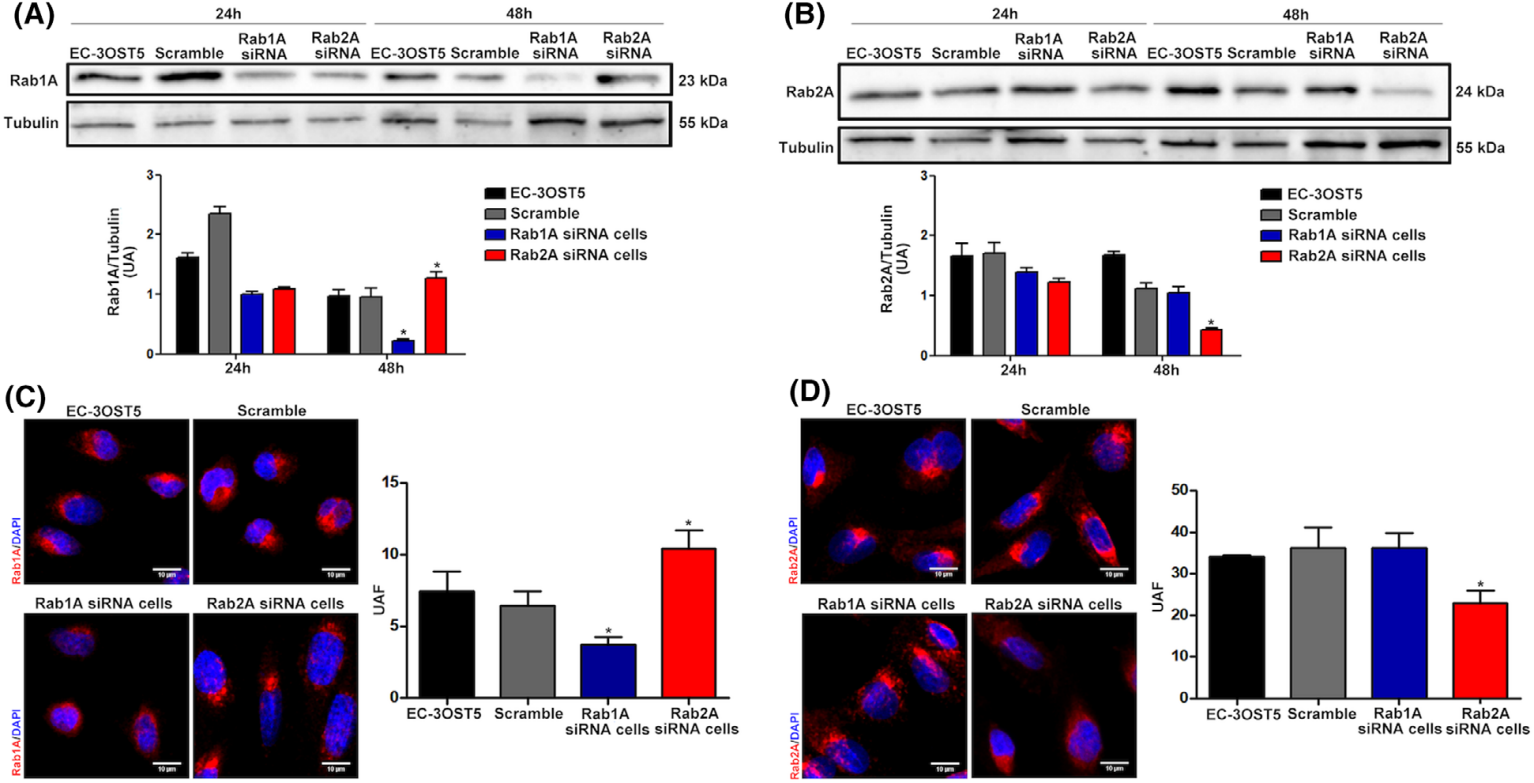
Protein profile of Rab1A and Rab2A in EC‐3OST5 after siRNA treatment. The protein expression of Rab1A and Rab2A was analysed over time (24 and 48 h) by western blotting (20 μg of protein) using specific antibodies to Rab1A (A) or Rab2A (B) in both siRNA transfected EC‐3OST5 cells. The ratio Rab1A/Tubulin and Rab2A/Tubulin (lower panels) corresponds to blot quantification performed in ImageJ software and displays the mean and standard deviation from two independent experiments. The expression profile of these proteins was also confirmed by confocal microscopy in cells with silenced Rab1A (C) and Rab2A (D) at 48 h. Rab1A and Rab2A are shown in red in their corresponding images and nuclei in blue. The right‐hand panels show the quantification of fluorescence in the red channel. The values were obtained in the Leica LAS X Life Science software, representing the mean and standard deviation of four images from two experiments. Scale bars: 10 μm. *P < 0.05, relative to control, scramble and between Rab proteins (One‐way ANOVA).

Despite their crucial role in organizing and maintaining the structure of the Golgi [19], the decrease in the expression levels of these two proteins did not affect the structure of the Golgi (Fig. 1H). A significant increase in Rab1A protein expression was observed in Rab2A siRNA cells (Fig. 2A,C), whereas there was no change in Rab2A protein expression in Rab1A knockdown cells (Fig. 2B,D), suggesting that a compensatory mechanism is at play.

### Rab1A and 2A are not directly linked to the expression of HS biosynthetic enzymes, but are linked to HS turnover

**Fig. 3:**
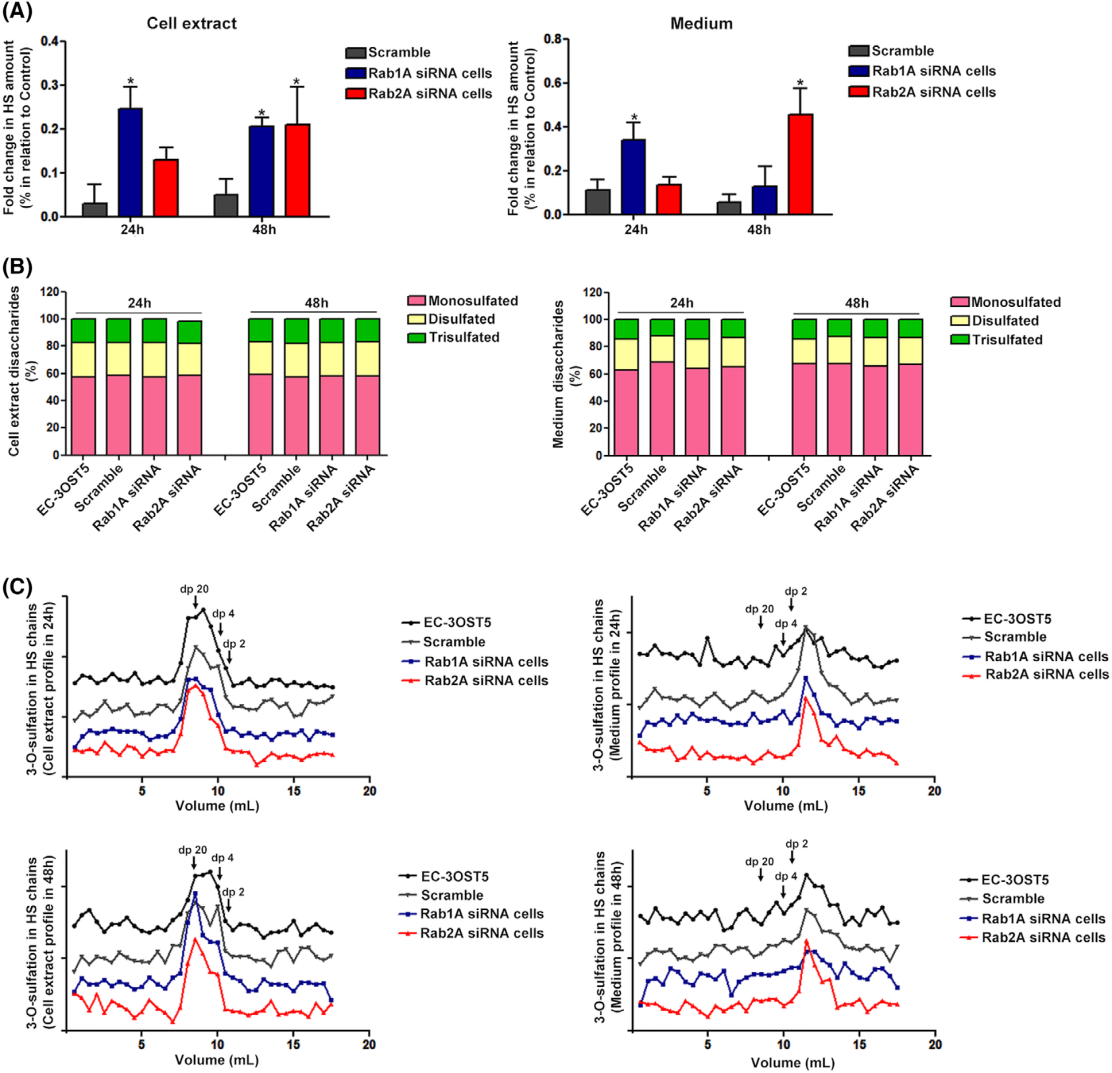
Synthesis and structural analysis of HS. (A) Quantification of HS present in the cell extract (left) and secreted into the culture medium (right) of EC‐3OST5 cells silenced for Rab1A and Rab2A at 24 and 48 h. The results represent the mean with SD from three independent experiments. (B) Disaccharide composition of HS purified from cells (left) and culture medium (right). The Δ‐disaccharides of HS were classified into mono‐, di‐ and trisulfated forms. (C) The size analysis of HS oligosaccharides containing 3‐O‐sulfation was conducted on the cell extract (right) and the medium (left) after 24 and 48 h (upper and lower panels, respectively). (B, C) The Δ‐degradation products of HS were generated from three independent experiments, and the digestion products were pooled before analysis to enable detection. The results represent an overall trend. *P < 0.05, relative to scramble vs Rab1A siRNA cells (24 and 48 h) and scramble vs Rab2A siRNA cells (48 h) in cell extract; relative to scramble vs Rab1A siRNA cells (24 h), scramble vs Rab2A siRNA cells (48 h) and Rab1A vs Rab2A (24 and 48 h) in culture medium (two‐way ANOVA). Dp denotes the degree of polymerization.

Rab1A knockdown cells showed a significant increase in the amount of HS in both the cell extract and secreted into the medium at 24 h; HS levels in cell extract remained higher at 48 h (Fig. 3A). This suggests that low levels of Rab1A expression may interfere with anterograde transport, promoting the accumulation and secretion of HS. On the other hand, the levels of HS remained unchanged in Rab2A knockdown cells after 24 h. At 48 h, there was a significant increase in the amount of HS in both the cell extract and the medium (Fig. 3A).

**Fig. 4:**
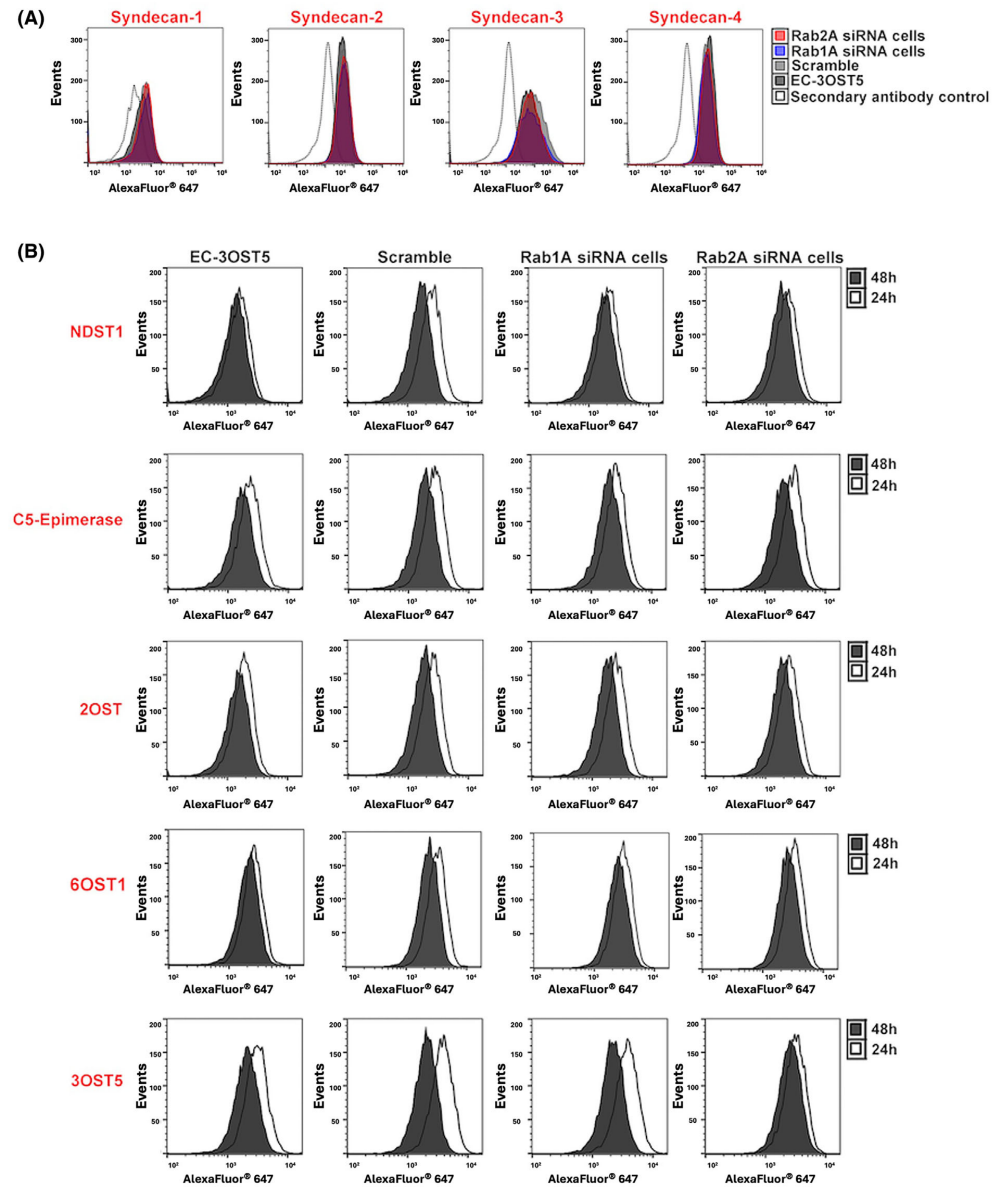
Protein expression of HSPGs and components of HS biosynthesis. Protein profile of HSPGs (A) and HS‐modifying enzymes (NDST1, C5‐epimerase, 6OST1, 2OST and 3OST5) (B) in Rab1A or Rab2A silencing cells was evaluated by flow cytometry using specific antibodies for each target protein. The expression of HS proteoglycans was evaluated at 48 h, while the expression of HS‐modifying enzymes was analysed at 24 h (colourless histogram) and 48 h (grey histogram). After incubating with primary antibodies, the cells were incubated with corresponding secondary antibodies conjugated with Alexa Fluor® 647 and then analysed in a BD Accuri C6 flow cytometer. The data illustrate the results from two independent experiments.

Fig. 3B shows no significant change in the disaccharide composition of HS amongst the different cells. The presence of 3‐O‐sulfation in HS chains was also analysed to complement the disaccharide analysis of HS. As the glycosidic linkage adjacent to 3‐O‐sulfated glucosamine is resistant to digestion, resulting in unsaturated oligosaccharides [20], HS chains were digested with heparinase II, and the products were analysed by size exclusion chromatography. Consistent with the previous HS structure results, there was also no change in this modification (Fig. 3C), and overall HS structure, as shown by the similar oligosaccharide profile produced (Fig. 3B,C), despite a slight decrease in the expression of enzymes responsible for the processes of N‐deacylation/N‐sulfation, O‐sulfation and epimerization (NDST1, C5‐epimerase, 2OST, 6OST1 and 3OST5) after 48 h after siRNA treatment (Fig. 4B). The protein expression of four syndecans remained unchanged in Rab1A and Rab2A knockdown cells (Fig. 4A).

### Rab1A and 2A mediate the localization of HS‐modifying enzymes

**Fig. 5:**
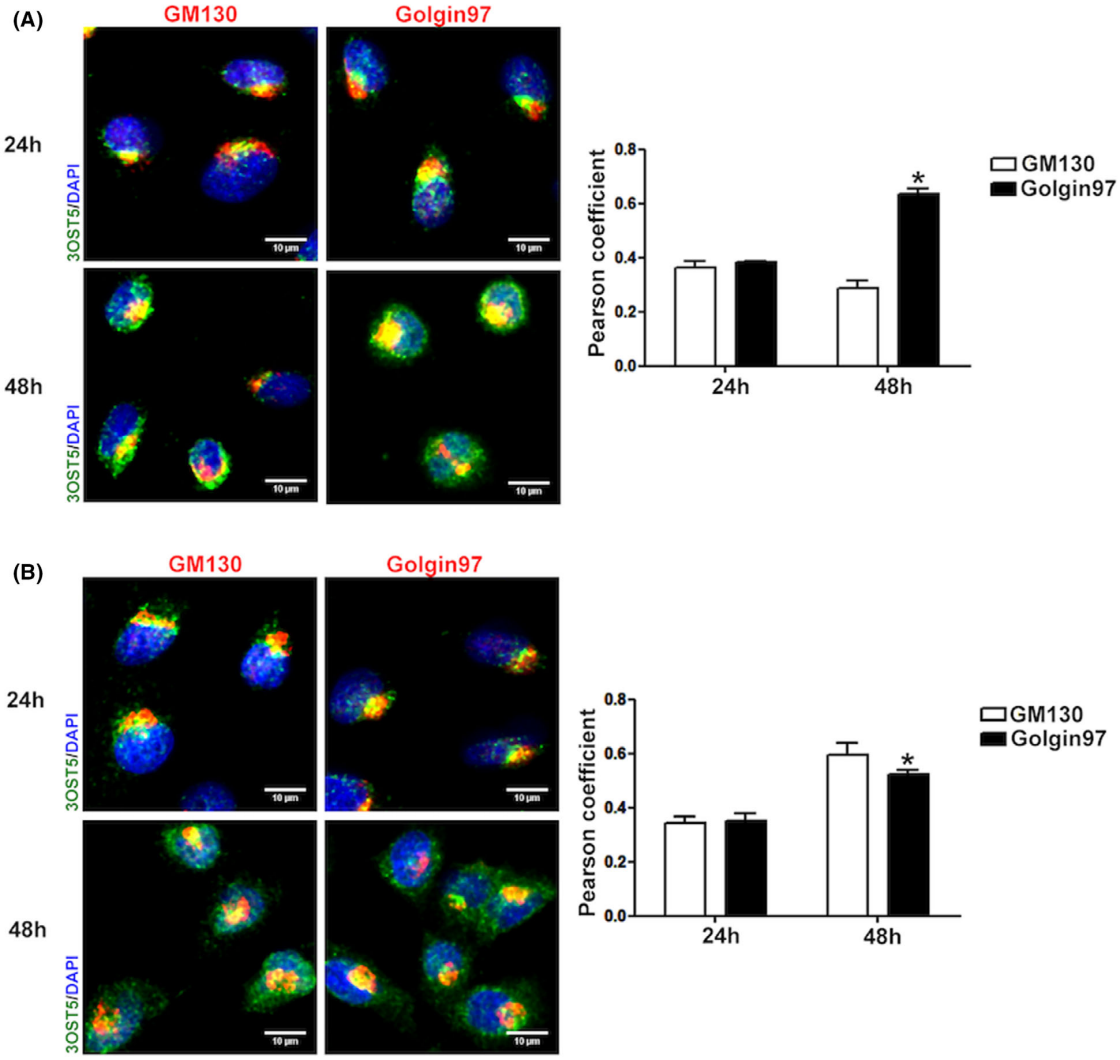
Subcellular localization of 3OST5 in Golgi compartments. After silencing the cells for Rab1A and Rab2A, EC‐3OST5 cells were double‐labelled with antibodies to GFP (tagged 3OST5, green) and specific antibodies to cis‐Golgi (GM130) and trans‐Golgi (Golgin97), both in red. Localization of 3OST5 in these different compartments of the Golgi was analysed in cells silenced for Rab1A (A) and Rab2A (B) for 24 and 48 h. Pearson's correlation coefficient represents the rate of co‐localization of recombinant 3OST5 in the different cellular components evaluated (right‐hand panels). The values were obtained in the Leica LAS X Life Science software, representing the mean and standard deviation of four images from two experiments. Scale bars in images: 10 μm. *P < 0.05, relative to GM130 vs Golgin97 in Rab1A and Rab2A siRNA cells (48 h, A, B) (Student's *t*‐test).

**Fig. 6:**
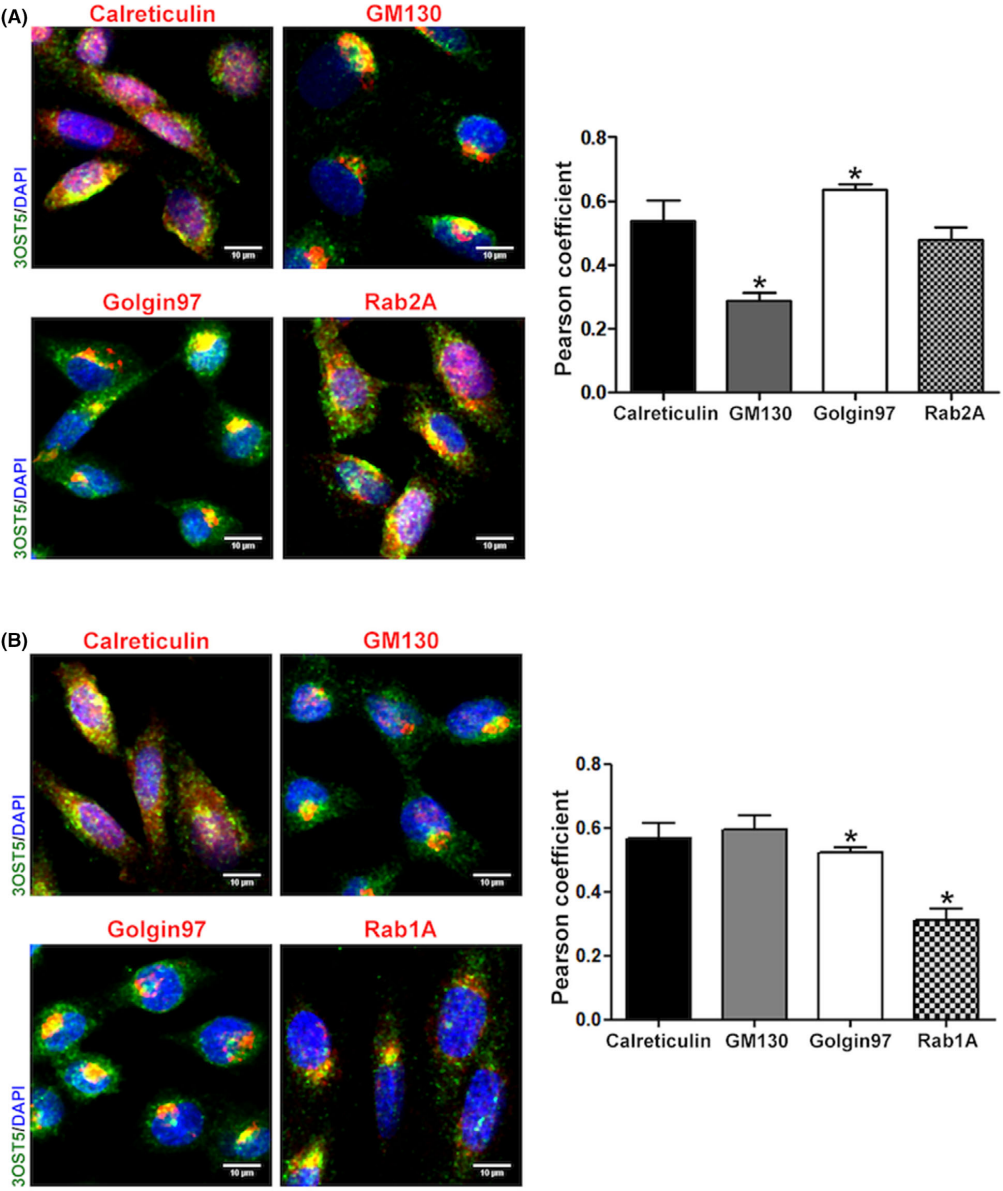
Subcellular localization of 3OST5 in the ER–Golgi pathway. The localization of 3OST5 in different cellular components involved in the ER–Golgi pathway was examined in cells where Rab1A (C) and Rab2A (D) were silenced for 48 h. Tagged 3OST5 is shown in green, whereas cis‐Golgi (GM130), trans‐Golgi (Golgin97), RER (Calreticulin) and Rab proteins are shown in red. Pearson's correlation coefficient represents the rate of colocalization of recombinant 3OST5 in the different cellular components evaluated (right‐hand panels). Data are presented as mean ± standard deviation of four images from two experiments. Scale bars in images: 10 μm. *P < 0.05, relative to GM130 and Golgin97 vs the other components in C; relative to GM130 vs Golgin97 and Rab1A vs the other components in D (One‐way ANOVA).

**Fig. 7:**
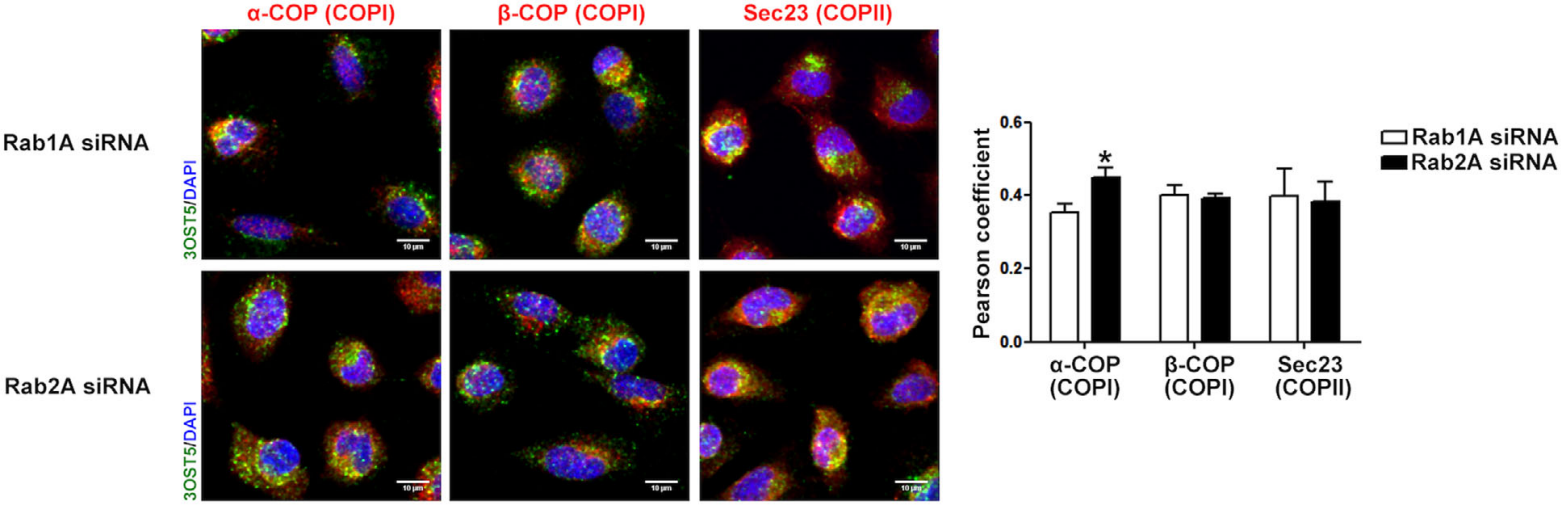
Subcellular localization of 3OST5 in coated vesicles. The distribution profile 3OST5 in coated vesicles was examined 48 h after siRNA treatment for Rab1A and Rab2A. COPI vesicles were visualized by α‐COP and β‐COP staining, and COPII vesicles were visualized by Sec23 staining. These cellular components are shown in red and tagged 3OST5 in green. Pearson's correlation coefficient represents the rate of colocalization of recombinant 3OST5 in the different coated‐vesicle components (right‐hand panels). Data are presented as mean ± standard deviation of four images from two experiments. Scale bars in images: 10 μm. *P < 0.05, relative to Rab1A vs Rab2A siRNA cells in α‐COP (Student's *t*‐test).

To further evidence the role of Rab1A and 2A in HS‐modifying enzyme trafficking, the localization of 3OST5 throughout the Golgi was investigated. The distribution of the 3OST5 between cis‐ and trans‐Golgi was similar in both silencing conditions at 24 h. Yet, a significant change in localization was observed in silenced cells at 48 h (Figs 5, 6, 7). While 3OST5 was predominantly located in trans‐Golgi in Rab1A‐silenced cells (Figs 5A and 6A), consistent with a reduction in transport towards the cis‐Golgi, in Rab2A knockdown cells, 3OST5 showed a preferential distribution in cis‐Golgi (Figs 5B and 6B), supporting impaired recycling of the enzyme in ER–Golgi pathway. Furthermore, the presence of significant 3OST5 levels in the cis‐Golgi compartment suggests the participation of other Rab proteins in COPI vesicle‐mediated transport. Finally, 3OST5 exhibited similar distribution in β‐COP, a subunit comprised of the F‐subcomplex of COPI, and in COPII vesicles, represented by staining of the Sec23 subunit. Nevertheless, in cells where Rab2A was silenced, 3OST5 exhibited greater colocalization with α‐COP, a subunit comprised of the B‐subcomplex of COPI and responsible for cargo recognition (Fig. 7).

### Involvement of the GOLPH3 protein in 3OST5 traffic

**Fig. 8:**
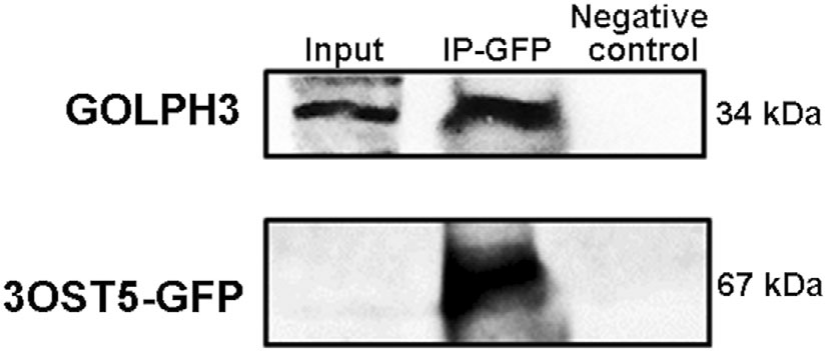
3OST5 interacts with GOLPH3. EC‐3OST5 cell lysate was immunoprecipitated for GFP using the anti‐GFP antibody and revealed for the GOLPH3 protein. The upper membrane was incubated with the primary antibody against GOLPH3 (1 : 1000) and detected using an anti‐rabbit HRP‐linked secondary antibody (1 : 2500). Meanwhile, the lower membrane was developed using only an anti‐goat HRP‐linked secondary antibody (1 : 2500) and, as a result, the band corresponding to the 3OST5‐GFP protein in the input lane is absent. The data reflect the outcomes of two independent experiments.

Owing to the fact that the GOLPH3 protein recognizes signals present in the cytoplasmic domain of many glycosyltransferases located in Golgi, including HS polymerization enzymes EXT1 and EXT2, and mediates their transport between Golgi cisternae via COPI [11, 17, 21, 22], we evaluated the role of this protein in the transport of 3OST5. As shown in Fig. 8, the immunoprecipitated complex was recognized by the anti‐GOLPH3, confirming the interaction between 3OST5 and GOLPH3 proteins. This suggests that the transport of HS‐modifying enzymes, here the 3OST5, via COPI vesicles, is mediated by GOLPH3. This is the first direct evidence of an HS‐modifying enzyme binding to a Golgi adaptor protein that is known to mediate cargo sorting and trafficking.

## Discussion

The complex substitution pattern inherent in HS chains encodes their ability to regulate numerous biological processes by interacting with hundreds of proteins [23]. N‐deacetylation/N‐sulfation, O‐sulfation and epimerization result from a sophisticated biosynthetic process that generates precise HS structures that modulate diverse physiological and pathological processes [24, 25, 26, 27].

While the polymerization and modification of HS chains demonstrate similarities across various organisms, the regulation of the biosynthetic process remains only partly understood [28]. Amongst the numerous factors influencing HS biosynthesis are enzyme actions occurring in either a sequential [7] or nonhierarchical manner [9, 10, 29], the formation of protein complexes [3, 4, 30, 31, 32], the localization of enzymes within the Golgi [11, 12] or in distinct Golgi compartments [33], diverse polysaccharide binding orientations [34, 35], and the involvement of transcription factors and microRNAs [36, 37, 38].

Recently, our research has uncovered the crucial role of HS‐modifying enzyme trafficking, mediated by COPI and COPII, in regulating HS biosynthesis. This process governs the spatial and temporal distribution of HS‐modifying enzymes across various Golgi cisternae, thereby influencing the composition of HS [12]. Building upon this concept, the objective of this study was to further explore the role of accessory protein components of vesicular transport in regulating HS biosynthesis. Specifically, we employed endothelial cells transfected with 3OST5 subjected to knockdown of Rab1A and Rab2A, involved in anterograde and retrograde vesicle‐mediated transport, respectively.

The findings indicate that silencing Rab1A and Rab2A proteins alters the temporal distribution of 3OST5 within Golgi compartments. In Rab1A‐silenced cells, 3OST5 predominantly localizes to the trans‐Golgi, suggesting reduced protein transport from the ER to the Golgi. Conversely, in Rab2A‐silenced cells, 3OST5 is primarily concentrated in the cis‐Golgi due to disrupted enzyme recycling between the Golgi and ER‐mediated by Rab2A. Additionally, reduced *de novo* protein synthesis is observed, as indicated by decreased HS‐modifying enzyme expression 48 h after siRNA treatment.

Silenced Rab1A cells demonstrated an increase in the total amount of HS content within the initial 24 h; no significant alteration was observed in disaccharide composition during the analysed time frame. By the 48‐h mark, a notable increase in HS content within the cell extract persisted, while HS levels in the medium reverted to baseline levels, suggesting a likely shift in HS turnover via endocytic vesicles; a process that is also regulated by Rab1A [39, 40, 41]. On the other hand, Rab2A‐silenced cells exhibited no significant change in HS structure within the first 24 h post silencing, nor in the amount of HS. However, a marked increase in HS content within both compartments was evident by the 48‐h mark following Rab2A knockdown. Interestingly, these cells displayed a significant alteration in Rab1A protein expression over time, indicating a modulatory relationship between Rab2A and Rab1A that may reflect a compensatory mechanism aimed at preserving cellular homeostasis and HS production; molecules that are both ubiquitous and key for normal cell function [1, 24]. Additionally, one may not rule out changes to trafficking/localization of additional HS biosynthetic enzymes. Yet, we have previously provided evidence that HS‐modifying enzymes may be sorted and trafficked by similar mechanisms [12].

Together, these findings suggest that Rab1A is a pivotal protein in regulating various stages of vesicular trafficking, including Golgi and/or lysosomal targeting, and may consequently be subject to additional regulatory mechanisms, such as modulation by Rab2A itself. Furthermore, the silencing of Rab1A and Rab2A did not lead to changes in protein expression levels of HS‐modifying enzymes and HS proteoglycans at 24 h, underscoring the critical role of their spatial distribution in the ER‐Golgi, rather than simply their expression profile, in HS biosynthesis. This further reiterates our previous observations that up‐ or downregulation of individual enzymes/proteoglycans alone may not explain changes in HS biosynthesis and fine structure [12].

Previous research has indicated that the recycling of Golgi glycosyltransferases relies on binding to GOLPH3. This adaptor protein interacts concurrently with β‐COP and δ‐COP, subunits of the COPI vesicle, and with the cytoplasmic tails of proteins earmarked for transport [17, 42, 43, 44]. This membrane trafficking is further governed by Rab1A, which interacts with GOLPH3, thereby regulating its distribution within the Golgi [45, 46]. In the context of HS biosynthesis, it has been demonstrated that the GOLPH3 protein interacts with EXT1 and EXT2, the polymerization enzymes of HS, thereby influencing their positioning across distinct Golgi cisternae and consequently affecting HS biosynthesis [11]. Building upon this, our study illustrates that the trafficking of 3OST5, an HS‐modifying enzyme, within COPI vesicles is also facilitated by the GOLPH3 protein. Additionally, one cannot rule out the possible role of HS chains in mediating cargo sorting and trafficking of its biosynthetic enzymes, but in the case of GOLPH3, there are no obvious HS‐binding sites on the surface of GOLPH3 [47]. Nonetheless, our results underline the role of GOLPH3 in sorting HS‐modifying enzymes along the RER–Golgi pathway, which, in part, may account for changes in the structure of HS. This is the first direct evidence that vesicle adaptor proteins bind to cargo responsible for fine‐tuning the HS sulfation pattern, here 3SOT5.

Collectively, the data presented illuminate novel mechanisms within vesicular transport by which HS biosynthesis is regulated, further reiterating the complexity of glycoconjugate biosynthesis. This complexity ensures that the regulation of the HS biosynthetic process and the structure of the HS produced is not reliant on a singular pathway but, rather, on multiple pathways, enabling swift and precise adjustments to the content and structure of HS when required to maintain homeostasis.

## Material and methods

### Reagents and antibodies

Fetal bovine serum, penicillin (10 000 U·mL^−1^), streptomycin (10 000 μg·mL^−1^) and Ham's F‐12 Nutrient Mixture were acquired from Gibco da Life Technologies Inc. (New York, NY, USA). G418 disulfate salt solution was purchased from Sigma Aldrich (Saint Louis, MO, USA). H_2_^35^SO_4_ carrier free was purchased from National Centre for Nuclear Research Radioisotope POLATOM (Otwock, Poland). HS oligosaccharides standards were purchased from Iduron (Alderley Edge, UK). Mouse antibodies against C5‐epimerase and Golgin97 were purchased from Abcam (Cambridge, MA, USA), antibody against NDST1 (M01) from Abgent (San Diego, CA, USA) and antibody against Rab2A from Origene (Rockville, MA, USA). Rabbit antibodies against α‐COP, β‐COP, GM130, Calreticulin and Syndecan‐3 were purchased from Abcam, antibodies against 3OST5 and COPII from Thermo Scientific (Rockford, IL, USA), antibody to 2OST (N‐term) from Abgent, antibodies against Rab1A and GOLPH3 from Origene and antibodies against 6OST1, Syndecan‐2 and Syndecan‐4 from Santa Cruz Biotechnology (Dallas, TX, USA). Goat antibody against GFP (I‐16) and Syndecan‐1 were obtained from Santa Cruz Biotechnology. Secondary antibodies conjugated to Alexa Fluor® 488, Alexa Fluor® 594 and Alexa Fluor® 647 were purchased from Thermo Fisher Scientific, while HRP secondary antibodies were obtained from Cytiva (Amersham, UK). Information regarding all these antibodies is specified in Table S1.

### siRNA and plasmids

Human Rab1A (Cat. no. SR303940) and human Rab2A (Cat. no. SR303941) siRNAs, composed of three unique 27mer siRNA duplexes each one, and scrambled negative control siRNA (Cat. no. SR3004) were purchased from Origene. The target sequences of human Rab1A siRNAs were the following: sequence A, forward: 5′‐CGAAUGUAGAACAGUCUUUCAUGAC‐3′ and reverse 5′‐GUCAUGAAAGACUGUUCUACAUUCGUU‐3′, sequence B, forward: 5′‐GCACUACAACAGAUUCUUACCGUCT‐3′ and reverse 5′‐AGACGGUAAGAAUCUGUUGUAGUGCAG‐3′, and sequence C, forward: 5′‐ GGAAGUAAUAUCAAACUGUAUGGTG ‐3′ and reverse 5′‐ CACCAUACAGUUUGAUAUUACUUCCAA ‐3′. As for the human Rab2A siRNAs, the sequences were: sequence A, forward: 5′‐GCUACAGUUUACAGACAAGAGGUTT‐3′ and reverse 5′‐AAACCUCUUGUCUGUAAACUGUAGCAA‐3′, sequence B, forward: 5′‐ACUACGAACUGAAUUGUAUUAAACA‐3′ and reverse 5′‐ UGUUUAAUACAAUUCAGUUCGUAGUUA‐3′, and sequence C, forward: 5′‐ GCUCGAAUGAUAACUAUUGAUGGGA‐3′ and reverse 5′‐ UCCCAUCAAUAGUUAUCAUUCGAGCAC ‐3′. Lastly, scrambled negative control siRNA sequences were: 5′‐CGUUAAUCGCGUAUAAUACGCGUAT‐3′ (forward) and 5′‐AUACGCGUAUUAUACGCGAUUAACGAC‐3′ (reverse). pAcGFP‐N1‐3OST5 expressing 3OST5‐GFP was prepared as described previously [12].

### Cell culture and siRNA knockdown of Rab proteins

Endothelial cells (ECs) derived from rabbit aorta [48] were transfected with pAcGFP‐N1‐3OST5 (EC‐3OST5) and characterized, including confirmation for EC markers, as described previously [12] and maintained in F12 medium supplemented with 10% (v/v) fetal bovine serum, penicillin (100 U·mL^−1^), streptomycin (100 μg·mL^−1^) and G418 disulfated salt (0.5 μg·mL^−1^) at 37 °C in a humidified atmosphere of 2.5% CO_2_. All cultures were confirmed not to be contaminated with mycoplasma by PCR. EC‐3OST5 cells were seeded with 40–50% confluence in 24‐well plates 1 day before siRNA transfection. The cells were transfected with 10 nm siRNA containing the mixture of three different sequences for Rab1A or Rab2A using Lipofectamine 3000 (Invitrogen, Carlsbad, CA, USA) according to the manufacturer's protocol. Two days later, the cells were analysed by qPCR, Flow cytometry and Western blotting.

### RNA extraction and quantitative real‐time PCR

Total RNA was extracted from siRNA transfected EC‐3OST5 cells using Trizol reagent (Invitrogen) following the manufacturer's instructions. Then, reverse transcriptase reaction was carried out with 2 μg of total RNA using ImProm‐II™ Reverse Transcription System (Promega, Madison, WI, USA) and aliquots of cDNA obtained were amplified in qPCRs, using the primers described in Table S2. Real‐time PCR amplifications were performed using Maxima® SYBR Green Master Mix 2X (Fermentas, Waltham, MA, USA). The PCR conditions were as follows: 95 °C for 10 min, followed by 40 cycles of 95 °C for 15 s and 60 °C for 60 s. The reactions were run in triplicate on the 7500 Real Time PCR System (Applied Biosystems, Waltham, MA, USA). Relative expression levels of genes were calculated using the $2^{-\Delta C_{t}}$ method and normalized to the housekeeping gene, COX IV (cytochrome *c* oxidase).

### Flow cytometry

siRNA transfected EC‐3OST5 cells were detached from the plate using citrate saline solution (135 mm KCl, 15 mm sodium citrate). Subsequently, the cells were fixed with 2% paraformaldehyde in PBS for 30 min and permeabilized with 0.01% saponin in PBS for an additional 30 min. Following this, the cells were incubated with primary antibody for 2 h, which was succeeded by incubation with fluorescent‐labelled secondary antibody for 40 min in the dark. Antibodies were diluted in PBS containing 1% BSA. Data acquisition was carried out using the BD Accuri C6 flow cytometer (BD Biosciences, Franklin Lakes, NJ, USA), and data analyses were performed using the flowjo v.10 software (Tree Star, Inc., Ashland, OR, USA).

### Western blotting

Cells were lysed in RIPA lysis buffer supplemented with Halt protease inhibitor cocktail (ThermoFischer Scientific) for 2 h on ice. Following clarification by centrifugation, protein concentration was assessed using BCA protein kit assay. Protein extracts (20 μg) were resolved by SDS/PAGE and transferred onto Hybond ECL Nitrocellulose membranes (Cytiva). After blocking with 5% fat‐free dried milk in Tris‐buffered saline, the membranes were incubated with primary antibodies at 4 °C overnight and incubated with HRP‐conjugated secondary antibodies at room temperature for 1 h. The signals were detected in UVITEC 4.7 Cambridge Image System using SuperSignal® West Pico Chemiluminescent Substrate (ThermoFischer Scientific).

### Immunofluorescence and confocal microscopy

EC‐3OST5 cells were seeded on 13‐mm coverslips placed in 24‐well plate and after siRNA transfection, the cells were fixed with 2% paraformaldehyde for 30 min, washed with 0.1 m glycine, incubated with permeabilization/blocking solution (0.02% saponin, 1% BSA in PBS solution, 30 min), labelled with primary antibodies for 2 h and then incubated with the appropriate fluorescent‐labelled secondary antibodies for 1 h at room temperature. Nuclei were stained with 4′,6‐diamidino‐2‐phenylindole (DAPI, Thermo Fischer Scientific, 1 μg·mL^−1^ in blocking buffer). Lastly, coverslips were mounted on glass microscope slides using a mounting medium (Fluoromount‐G) and images were captured on a Leica TCS SP8 CARS confocal microscope (Wetzlar, Germany) with HC PL APO 63×/1.40 oil immersion objectives. The images represent the sum of slide projections corresponding to the z‐series of confocal stacks. Pearson's correlation values of fluorescence images were obtained in Leica LAS X Life Science software (Leica Microsystems, Wetzlar, Germany).

### Composition analysis of HS disaccharides

PGs synthesized by the siRNA transfected EC‐3OST5 cells were metabolically labelled with carrier free [^35^S]‐sulfate (150 μCi·mL^−1^) in serum‐free F12 medium (24 h, 37 °C) in a humidified atmosphere with 2.5% CO_2_. After labelling, the culture‐conditioned medium was collected, and the cells were removed from the plate with 3.5 m urea in 25 mm Tris–HCl pH 8.0. After proteolysis with papain (1 mg·mL^−1^ in phosphate‐cysteine buffer, 60 °C for 18 h), nucleic acids and peptides were precipitated using trichloroacetic acid (TCA 90%) and the GAGs in the supernatant were precipitated by adding three volumes of iced methanol at −20 °C for 24 h. The precipitate was collected by centrifugation, dried and suspended in distilled water. The sulfated GAGs were then characterized and quantified by agarose gel electrophoresis in PDA buffer (0.05 m 1,3‐diaminepropane acetate) [49]. The analysis of the disaccharide composition of the HS extracted was carried out by enzymatic degradation with heparinases II and III from *Flavobacterium heparinum* followed by liquid chromatography using a PhenoSphere™ 5 μm SAX (150 × 4.6 mm, Phenomenex) column as described previously [4]. The Δ‐degradation products of HS were expressed as monosulfated, disulfated and trisulfated disaccharide groups. The results represent the media of three independent experiments.

### Analysis of 3‐O‐sulfated oligosaccharides

The evaluation of the presence of 3‐O‐sulfation in HS chains was performed by incubation [^35^S]‐HS extracted described above with heparinase II from *Flavobacterium heparinum* followed by size exclusion chromatography [20]. After incubation of [^35^S]‐HS chains with 40 μL of heparitinase II in 20 mm Tris–HCl, pH 7.4 containing 4 mm CaCl_2_ e 50 mm NaCl at 30 °C for 18 h, the ^35^S‐labelled degradation products were analysed in a Yarra Sec‐2000 (300 × 7.8 mm, Phenomenex) column, previously calibrated with HS oligosaccharides standards (dp20, dp4 and dp2) (Fig. S2), in PBS buffer for 30 min at a flow rate of 0.5 mL·min^−1^. Individual fractions (0.5 mL) were collected and counted in 1 mL of Ultima Gold (PerkinElmer, Waltham, MA, USA) on a Micro‐Beta counter.

### Co‐immunoprecipitation assay

EC‐3OST5 cells were lysed in IP buffer (150 mm NaCl, 50 mm Tris–HCl pH = 7.5, 1% NP‐40, 2 mm EDTA and Halt protease inhibitor cocktail) for 30 min on ice and insoluble materials were removed by centrifugation at 10 000 ***g*** for 30 min at 4 °C. After a preclearing step by incubation 1 mg of protein extract with 30 μL protein G Plus‐agarose (Santa Cruz Biotechnology) in 1 mL of IP buffer for 1 h, the cleared lysate was incubated with 80 μL protein G, previously complexed to 1 μg anti‐GFP polyclonal for 4 h at 4 °C on the wheel, for 18 h at 4 °C. After two washes with 1 mL of IP buffer and one wash with 1 mL PBS containing protease inhibitors, the immunoprecipitated complexes were resuspended in 40 μL of Laemmli sample buffer 4× and were analysed by Western blotting using anti‐GOLPH3 antibody.

### Statistical analysis

The statistical analysis was conducted using either one‐way analysis of variance (ANOVA) followed by the Turkey test, two‐way ANOVA, or Student's *t*‐tests, depending on the experiments. Statistical significance was established at *P* < 0.05.

## Conflict of interest

The authors declare no conflict of interest.

## Author contributions

MCZM was involved in conceptualization, methodology, investigation, writing—original draft, writing—review & editing. RPC was involved in investigation. EAY was involved in conceptualization, writing—review & editing, supervision. HBN was involved in writing—review & editing, resources, supervision, project administration, funding acquisition. MAL was involved in conceptualization, methodology, writing—review & editing, supervision, project administration, funding acquisition.

### Peer review

The peer review history for this article is available at https://www.webofscience.com/api/gateway/wos/peer‐review/10.1111/febs.17398.

## Supporting information


**Fig. S1.** Characterization of EC‐3OST5 cells.
**Fig. S2**. Size exclusion chromatography of heparan sulfate oligosaccharide standards.
**Table S1**. Sets of antibodies used in this study.
**Table S2**. Sets of primers used in real‐time PCR.

## Data Availability

The data sets generated and analysed during this study are available from the corresponding author upon reasonable request.
