# Supplementary material for: Involvement of GTPases and vesicle adapter proteins in Heparan sulfate biosynthesis: role of Rab1A, Rab2A and GOLPH3
Source: FEBS J. 2025 Jan 13;292(9):2237–50. doi: 10.1111/febs.17398 (PMC12062774; doi:10.1111/febs.17398)
Supplement: Supplementary file 1 — Fig. S1. Characterization of EC‐3OST5 cells. Fig. S2. Size exclusion chromatography of heparan sulfate oligosaccharide standards. Table S1. Sets of antibodies used in this study. Table S2. Sets of primers used in real‐time PCR. [file FEBS-292-2237-s001.pdf]

Involvement of GTPases and Vesicle Adapter Proteins in Heparan Sulfate Biosynthesis: role of Rab1A, Rab2A, and GOLPH3.

Maria C.Z. Meneghetti[1*], Renan P. Cavalheiro[1], Edwin A. Yates[2,1], Helena B. Nader[1] & Marcelo A. Lima[3,1*]

[1]Departamento de Bioquímica, Instituto de Farmacologia e Biologia Molecular, Escola Paulista de Medicina, Universidade Federal de São Paulo, Rua Três de Maio, 100 – São Paulo, SP, 04044-020, Brazil

[2]Department of Biochemistry, Cell and Systems Biology, Institute of Systems, Molecular and Integrative Biology, University of Liverpool, Liverpool, Liverpool L69 7ZB, UK

[3]Centre for Glycoscience, Keele University, Keele, Staffordshire, ST5 5BG UK

***Corresponding author:**

Maria C.Z. Meneghetti

mcz.meneghetti@unifesp.br

Marcelo A. Lima

m.andrade.de.lima@keele.ac.uk

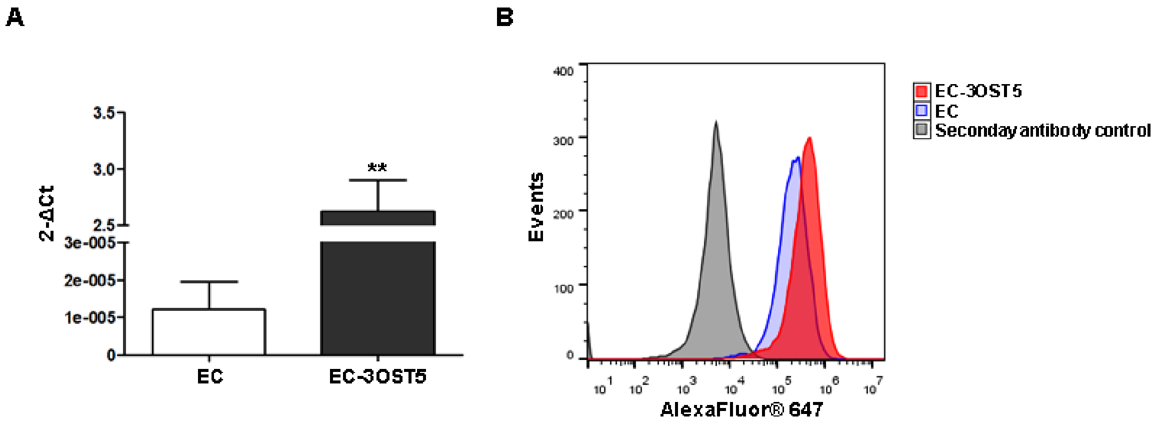

Fig. S1. Characterization of EC-3OST5 cells. (A) 3OST5 mRNA level in EC and EC-3OST5 cells was analyzed by real-time PCR. The values represent the mean and standard deviation of the tests carried out in triplicates. (B) The total level of 3OST5 protein expression was assessed by flow cytometry using the anti-3OST5 antibody (1:50). Following incubation with the primary antibody, the cells were incubated with anti-rabbit Alexa Fluor® 647 secondary antibody (1:200) and analyzed using a BD Accuri C6 flow cytometer. **$p<0.05$, relative to EC (Student´s t-test).

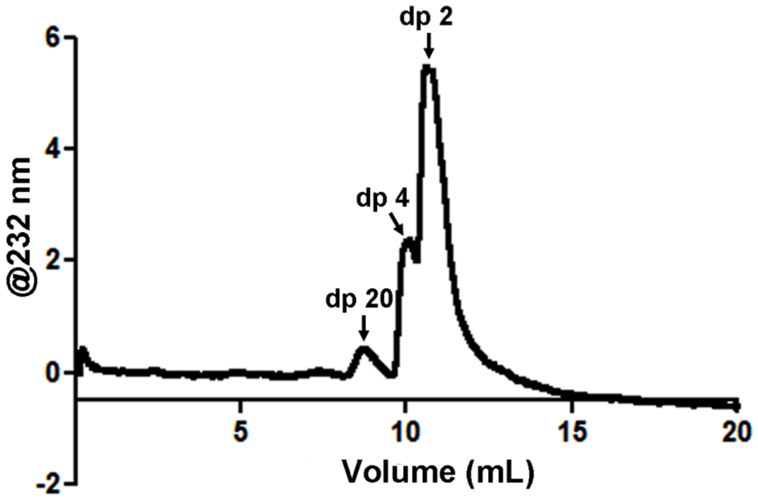

Fig. S2. Size exclusion chromatography of HS oligosaccharide standards. The Yarra Sec-2000 column was previously calibrated with HS oligosaccharide standards of different degrees of polymerization (dp): 20, 4 and 2. The samples are eluted in PBS buffer for 30 min at a flow rate of 0.5 mL/min and detected at a wavelength of 232 nm in ÄKTA Pure™ chromatography system. Dp denotes degree of polymerisation.

Table S1. Sets of antibodies used in the present study.

Antibodies	Dilution	Host	Clonality	Company
GFP	Immunofluorescence (1:250)<br>IP (1 μg)	Goat	Polyclonal	Santa Cruz Biotech<br>(sc-5385)
Rab1A	Immunofluorescence (1:200)<br>Flow cytometry (1:50)<br>Western blotting (1:1000)	Rabbit	Polyclonal	Origene<br>(TA342210)
Rab2A	Immunofluorescence (1:350)<br>Flow cytometry (1:50)<br>Western blotting (1:1000)	Mouse	Polyclonal	Origene<br>(TA347035)
α-COP	Immunofluorescence (3 μg/mL)	Rabbit	Polyclonal	Abcam<br>(cat ab2913)
β-COP	Immunofluorescence (1:1000)	Rabbit	Polyclonal	Abcam<br>(cat ab2899)
COPII (Sec23)	Immunofluorescence (1:200)	Rabbit	Polyclonal	Thermo Scientific<br>(cat PA1-069A)
GM130	Immunofluorescence (1:200)	Rabbit	Monoclonal	Abcam<br>(cat ab52649)
Golgin97	Immunofluorescence (5 μg/mL)	Mouse	Polyclonal	Abcam<br>(cat ab169287)
Calreticulin	Immunofluorescence (1:200)	Rabbit	Polyclonal	Abcam<br>(cat ab2907)
NDST1 (M01)	Flow cytometry (1:50)	Mouse	Monoclonal	Abgent<br>(cat AT2995a)
C5-Epimerase	Flow cytometry (1:50)	Mouse	Polyclonal	Abcam<br>(cat ab68714)
2OST (N-term)	Flow cytometry (1:50)	Rabbit	Polyclonal	Abgent<br>(cat AP7648a)
6OST1	Flow cytometry (1:50)	Mouse	Monoclonal	Santa Cruz Biotech<br>(sc-398231)
3OST5	Flow cytometry (1:50)	Rabbit	Polyclonal	Thermo Scientific<br>(cat PA5-54522)
Syndecan-1	Flow cytometry (1:50)	Goat	Polyclonal	Santa Cruz Biotech<br>(sc-7099)
Syndecan-2	Flow cytometry (1:50)	Rabbit	Polyclonal	Santa Cruz Biotech<br>(sc-15348)
Syndecan-3	Flow cytometry (1:50)	Rabbit	Polyclonal	Abcam<br>(ab63932)
Syndecan-4	Flow cytometry (1:50)	Rabbit	Polyclonal	Santa Cruz Biotech<br>(sc-15350)
GOLPH3	Western blotting (1:1000)	Rabbit	Polyclonal	Origene<br>(TA306858)
Anti-goat AlexaFluor® 488	Immunofluorescence (1:250)	Donkey	Polyclonal	Molecular Probes<br>(cat: A11055)
Anti-goat AlexaFluor® 647	Flow cytometry (1:200)	Donkey	Polyclonal	Molecular Probes<br>(cat: A21447)
Anti-mouse AlexaFluor® 594	Immunofluorescence (1:250)	Goat	Polyclonal	Molecular Probes<br>(cat A11005)
Anti-mouse AlexaFluor® 647	Flow cytometry (1:200)	Donkey	Polyclonal	Molecular Probes<br>(cat A31571)
Anti-rabbit AlexaFluor® 594	Immunofluorescence (1:250)	Goat	Polyclonal	Molecular Probes<br>(cat A11009)
Anti-rabbit AlexaFluor® 647	Flow cytometry (1:200)	Goat	Polyclonal	Molecular Probes<br>(cat A21245)
Anti-rabbit HRP-linked	Western blotting (1:2500)	Donkey		Cytiva<br>(NA934V)
Anti-mouse HRP-linked	Western blotting (1:2500)	Sheep		Cytiva<br>(NXA931)
Anti-goat HRP-linked	Western blotting (1:2500)	Rabbit	Polyclonal	Sigma-Aldrich<br>(A8919)

Table S2. Sets of primers used in real-time PCR.

Sense (S) e Antisense (AT) primers (5' - 3')		
**COX IV**	S:	GACGAGAAGGTCGAGTTGTATC
	AT:	CGGTGAAGCCGATGAAGAA
**3OST5**	S:	CTGCTTGAAATGCTGAACCTAC
	AT:	ATACCACTCAATGCCCTTACC
**RAB1A**	S:	GTCCAGCATGAATCCCGAATA
	AT:	GCAAACCTAAGAAGAAGGCAAG
**RAB1B**	S:	GCAGGAGATTGACCGCTATG
	AT:	TGTTGTCCACCACCTTCTTG
**RAB2A**	S:	GTACATCATAATCGGCGACACA
	AT:	GCACCGAACTCTACACCAATAG
**RAB2B**	S:	CTGTCCACGACCTCACAATAG
	AT:	TGTAGTAGGAACGGGTGATAGA
**RAB6A**	S:	CCCAGTTACATCCGTGATTCT
	AT:	GTTCTGACATCATCAATCCACTTT
